# Editorials

**Published:** 1897-07

**Authors:** 


### HOMŒOPATHIC PHYSICIAN,

A MONTHLY JOURNAL OF

HOMŒOPATHIC MATERIA MEDICA AND CLINICAL MEDICINE.

"If our school ever gives up the strict inductive method of Hahnemann, we are lost, and deserve only to be mentioned as a caricature in the history of medicine."—CONSTANTINE HERING.

Vol. XVII. JULY, 1897. No. 7.

### EDITORIALS.

CALCAREA-CARBONICA.—One of the characteristics of this remedy is a delirium resembling delirium tremens with delirious talk about rats and mice, fire and murder. It is often indicated in delirium tremens. Lachesis is indicated in delirium tremens when the patient develops great loquacity, jumping from subject to subject, and cannot bear to have anything touch the neck.

Calcarea has congestion of blood to the head like Belladonna. Calcarea is worse in the morning, when awaking, while Belladonna is better from sleep. Zinc has congestion to the head, worse from small quantities of wine. Conium has intoxication from small quantities of wine. These notes recall the notes made under Conium in the editorial in the January number.

Calcarea has headache from overlifting. This is a characteristic.

Calcarea has aggravation of headache by mental exertion. This, too, is a characteristic.

Calcarea has headache, ameliorated by closing the eyes and lying down.

Belladonna has the reverse—worse from closing the eyes and lying down.

Petroleum, every mental exertion causes him to become quite stupid.

This condition of mental exertion suggests a number of comparisons. Here they are; some derived directly from Dr. Lippe, and some from other sources, mainly, Dr. Hering:

Mental exertion causes sense of helplessness from brain weakness; can't attend to anything requiring thought, Gelsemium.

Torn feeling in the throat during mental exertion, Conium and Causticum.

Can't fix his mind on his studies, Iris-vers.

Mental exertion causes hemorrhage of the lungs, Rhus-tox.

Mental exertion causes pain in the occipital region, Picric-acid.

Mental exertion aggravates headache and produces sleep, Spigelia.

Mental exertion aggravates headache, Glon., Nat-mur., Nux-v., Spigel.

Mental exertion causes headache, Aur-m., Glon.

Mental exertion causes pressing pains in the head, Argent-nit.

Mental exertion, if excessive, causes irritation of the stomach, Nux-moschata.

Mental exertion aggravates pressing, boring headache, Colch., Nux-v.

Mental exertion aggravates mental symptoms, Nux-vom.

Mental ailments caused by mental exertion, Nux-vom.

Inability for mental exertion, Sepia. Compare with Gels., above.

Bad effects of excessive mental exertion in bookkeepers and others of similar pursuits, Anacardium, Sepia.

Mental exertion causes fullness in the head, intense headache, throbbing felt deep in the brain, and pain in left temple, Psorin.

Mental exertion causes aggravation in general, Aurum-met., Calc-c., Carbo-v., Colch., Mag-carb.

Mental exertion causes aggravation of various pains, especially hemorrhoids, Causticum.

Mental exertion causes bruised soreness all over the head, China.

Mental exertion causes sense of bruised soreness in the brain, China.

Mental exertion aggravates the headache that follows suppressed coryza, China.

Mental exertion causes him to become stupid with headache in the forehead, Petroleum.

Mental exertion causes pressure in the head, especially in the occiput; also deep in the cerebellum, Colch.

Mental exertion, when exhausting, aggravates pressing pain on top of head and in left frontal bone, with running together of letters when reading, Argent-nit.

Mental exertion, or fixing the thoughts on anything, is impossible; on attempting it has darkness before the eyes and increased headache, Argent-nit.

Mental exertion, if moderate, improves many ailments, Ferrum.

---

Vital Force.—The excellent paper bearing this title, written by the venerable Dr. J. M. Selfridge, of Oakland, California, and published in the June number of this journal, at page 188, was read before the California State Society. It created quite a sensation, revealing, as it did, the wide field of reading of its distinguished author in the domain of physical science. The Society recognized its merit by giving him a vote of thanks, while the members individually congratulated him.

We take the opportunity of joining with the members of the Society in offering Dr. Selfridge our own congratulations.

